# Tendon and multiomics: advantages, advances, and opportunities

**DOI:** 10.1038/s41536-021-00168-6

**Published:** 2021-10-01

**Authors:** Paula Sarmiento, Dianne Little

**Affiliations:** 1 Weldon School of Biomedical Engineering, Purdue University, West Lafayette, IN USA; 2 Department of Basic Medical Sciences, Purdue College of Veterinary Medicine, Purdue University, West Lafayette, IN USA

**Keywords:** Tissue engineering, Regenerative medicine, Proteomics

## Abstract

Tendons heal by fibrosis, which hinders function and increases re-injury risk. Yet the biology that leads to degeneration and regeneration of tendons is not completely understood. Improved understanding of the metabolic nuances that cause diverse outcomes in tendinopathies is required to solve these problems. ‘Omics methods are increasingly used to characterize phenotypes in tissues. Multiomics integrates ‘omic datasets to identify coherent relationships and provide insight into differences in molecular and metabolic pathways between anatomic locations, and disease stages. This work reviews the current literature pertaining to multiomics in tendon and the potential of these platforms to improve tendon regeneration. We assessed the literature and identified areas where ‘omics platforms contribute to the field: (1) Tendon biology where their hierarchical complexity and demographic factors are studied. (2) Tendon degeneration and healing, where comparisons across tendon pathologies are analyzed. (3) The in vitro engineered tendon phenotype, where we compare the engineered phenotype to relevant native tissues. (4) Finally, we review regenerative and therapeutic approaches. We identified gaps in current knowledge and opportunities for future study: (1) The need to increase the diversity of human subjects and cell sources. (2) Opportunities to improve understanding of tendon heterogeneity. (3) The need to use these improvements to inform new engineered and regenerative therapeutic approaches. (4) The need to increase understanding of the development of tendon pathology. Together, the expanding use of various ‘omics platforms and data analysis resulting from these platforms could substantially contribute to major advances in the tendon tissue engineering and regenerative medicine field.

## Introduction

Tendinopathy results in pain and hard to heal, debilitating orthopaedic conditions^1^. Tendinopathy ranges across a spectrum of acute, chronic, and acute-on-chronic degeneration leading to micro- or macro-scale tears that sometimes progress to rupture^2^. These conditions continue to be a medical and surgical management challenge because tendons heal by fibrosis and not by regeneration^3^. For instance, around 250,000 rotator cuff tears are repaired in the U.S. each year, and while surgical repair is cost effective, re-tear rates of up to 90% for rotator cuff repairs are reported^4–6^, suggesting that much works remain to be done to improve outcomes.

Regenerative medicine and tissue engineering strategies aim to improve tendon healing. However, numerous factors play a role in these healing microenvironments, and for example, analysis of all the possible biological and microarchitectural variables to determine the most successful healing strategy is exceedingly difficult. The number of changes to the healing tendon and its complex extracellular matrix (ECM) resulting from regenerative strategies can only be partially characterized using traditional molecular approaches, thus making comparisons between strategies somewhat difficult. This is one example where emerging fields such as multiomics could improve research processes and improve the likelihood of successful translation.

**Fig. 1:**
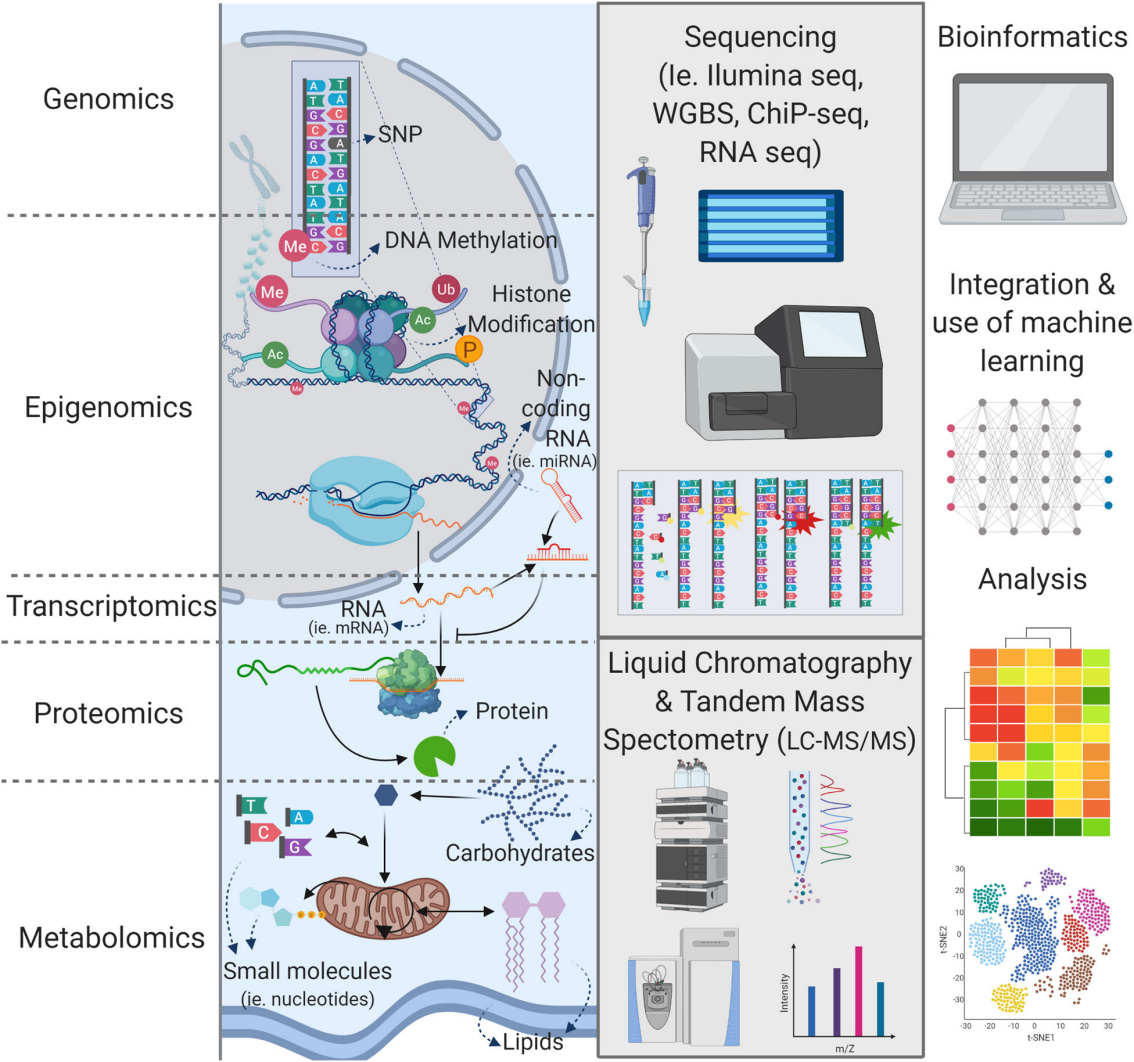
Individual ‘omics techniques, schematic description of their targets, and techniques used to study them. ‘Omics encases different subfields that are part of overall tissue metabolism: genomics, epigenomics, transcriptomics, proteomics, and metabolomics. Genomics studies the DNA sequence in the sample and identifies anomalies (e.g., single nucleotide polymorphisms (SNPs)) and is commonly achieved through whole-genome sequencing. Epigenomics refers to different factors that affect gene expression and uses different techniques according to the specific factors. For instance, DNA methylation is identified through whole-genome-bisulfite sequencing (WGBS), Histone modification is studied through chromatin immunoprecipitation sequencing (ChIP-Seq) and non-coding RNA is quantified through total RNA and small RNA sequencing (RNA-Seq). Transcriptomics studies the resulting gene expression and normally refers to the quantification of sequenced coding biotypes of the RNA, unless non-coding or small components are specifically included at the RNA isolation stage. Proteomics studies the protein profiles resulting from transcriptional regulation and translation, the identification of proteins is done through LC-MS/MS of digested tissue samples. Metabolomics studies small molecules that are components of metabolic processes including polar (i.e., nucleotides, ATP, carbohydrates) and non-polar (i.e., triglycerides, hormones, phospholipids), or lipidomic phases. Each metabolomic phase is isolated and then analyzed through LC-MS/MS. Multi-omics then, refers to the data integration of these different techniques by the enrichment of the data integrating the biological pathways and extraction of mechanistic information that can be obtained through these techniques. Machine learning techniques including deep learning or classification techniques are bioinformatics approaches to identify the difference in metabolism between groups, or to categorize the samples according to their similarity. Created in Biorender.com.

The term multiomics describes the aggregation of several genome-wide scale multidisciplinary fields and high throughput scientific platforms, each individually named with their own -omic suffix. Each ‘omic platform focuses on a specific link in molecular biology’s central dogma: genomics, metagenomics, transcriptomics, proteomics, and metabolomics (Fig. 1), and in some cases, each platform can also be sub-divided into ‘bulk’ (tissue-wide), and ‘spatial’—regional, single-cell, or sub-cellular approaches (e.g., nuclear or mitochondrial). Thus, core features of multiomic studies are that there is no need for a priori definition of study molecular targets, and individual samples can be wholly and multi-dimensionally characterized in a single experiment. Since multiomics identifies biomolecular profiles that link genotype to phenotype via the study of genetic material, proteins, and metabolites (Fig. 1), these platforms are helpful to understand, compare and define phenotypic states (i.e., healthy or diseased), and to follow phenotypic transitions (i.e., cell programming, development, or treatment response)^7^. As such, each of the sub-fields and platforms focuses on different components of the overall cell and tissue metabolism, which, when combined, build a more complete image than evaluation of a single ‘omic platform and alludes to the reasons for these phenotypic changes^8–10^. Therefore, following validation of putative molecular or metabolic targets derived from multiomic analyses, this enhanced understanding of both upstream and downstream events could ultimately lead to the development of new strategies to improve patient outcomes.

Multiomics present an immense opportunity for tendon research and regenerative approaches. Yet, there are relatively few studies in the field that have used these tools. This review aims to identify those studies and highlight their findings while identifying knowledge gaps pertaining to tendon biology and regeneration using multiomics techniques, gaps that once filled could improve current treatments for tendon healing and regeneration. We review the advances in the field from individual ‘omic techniques and the insights this has provided into four areas critical to tendon characterization: biology, degeneration and healing, in vitro phenotype, and regenerative and therapeutic approaches in vitro and in vivo.

## Discussion of literature

### Biology of the tendon

The complexity of tendon biology and the number of variables and factors that affect the biology necessitate a streamlined approach by comparing no more than 1- or 2-variables at any time within a given experiment or dataset. However, it is critical, especially if results are unexpected, or when comparing between studies to critically evaluate other characteristics that could play a role—accurate reporting of all relevant factors is therefore crucial.

#### Regional heterogeneity

Interpreting ‘omic profiles of tendon is challenging because of the complexity of the tissue and its heterogeneity, both within individual tendons and across different anatomic sites and functions. For example, the Achilles tendon is highly anisotropic, whereas the supraspinatus tendon has both highly anisotropic regions and more isotropic regions. While the main function of each is to buffer and transmit strain^11^, the biomechanics of each anatomic location is different, and this is reflected in their hierarchical fiber organization and likely in their response to mechanical stimuli by stratified matrix deposition^12^, although this has not yet been fully characterized across all tendons commonly impacted by tendinopathies. Importantly however, these differences likely impact both ‘bulk’ and ‘regional’ or spatial ‘omics outcomes, and several investigators have already begun to explore these differences in several tendon regions. Enhanced understanding of regional heterogeneity will improve understanding of structure-function relationships, and ultimately, to improved engineered tendon opportunities.

In mouse Achilles, following isolation by sequential enzymatic digestion, progenitors from the tendon proper are enriched in Scleraxis (*Scx*) and Tenomodulin (*Tnmd*), whereas those derived from the peritenon are enriched in vascular and pericyte markers including CD-133^13^. RNA-Seq data extended the list of enriched genes for tendon proper to include homeobox protein mohawk (*Mkx*), fibromodulin (*Fmod*), Thrombospondin 4 (*Thbs4*), Growth and differentiation factor 5 (*Gdf5*), and Wnt family Member 10a (*Wnt10a*). Conversely, markers for peritenon were extended to include markers more typical of a vascularized tissue, collagen 15 alpha 1 (*Col15a1*), integrin subunit alpha-4 (*Itga4*), a disintegrin and metalloproteinase with thrombospondin motifs (*Adamts16*), and a marker of endothelial and white blood cell lineages, fibrinogen like 2 (*Fgl2*)^13^. In addition, an analysis of the core and periphery of equine tendons showed substantial overlap, but also nuances in ECM protein expression between different load-bearing regions of the same tendon^14^, although the peripheral tendon evaluated in this study also included some peritenon. Type IV collagen (COL4A2) was more abundant in cultures of the core tendon, whereas lysyl oxidase-like 2 (LOXL2), fibulin 5 (FBLN5), and a disintegrin and metalloproteinase domain-containing protein 10 (ADAM10) were more abundant in the periphery compared to the core. Longitudinally, through the muscle-tendon-bone unit, the transition from muscle to tendon at the myotendinous junction was elaborated via proteomic analyses in the mouse soleus. Proteins related to the basal lamina were found primarily in the muscle, and proteins related to the tendon ECM as tenascin-C (*Tnc*) were more abundant in the tendon. In addition, the junction showed characteristics of both muscle and tendon, but some proteins, for example, *Col22A1* were restricted to the junction^15^. Similarly, single-cell analysis of tendon-to-bone attachment or enthesis, showed clear demarcation between tendon and cartilage to cells at the attachment itself^16^. These cells express a mix of both tenocyte and cartilage transcriptomic profiles. In addition, this transitional region expresses transcription factors, such as the Krüppel-like factors (KLFs), *Lmo1*, and *Gli1*, that may be the master regulators for this cell cluster. Furthermore, it was demonstrated that this region is composed of bi-fated cells that accordingly share a mix of accessible chromatin regions^16^.

Single-cell transcriptomics has also elucidated differences within the tendon. De Micheli et al.^17^ identified 13 cell types in the mouse Achilles tendon, where four groups correspond to tenocytes, and the remaining corresponds to vasculature, signaling, nervous tissue and pericytes. Three of the tenocyte groups expressed unique ECM binding genes: secreted phosphoprotein 1 (*Spp1*), dermatopontin (*Dpt*), and SPARC-related modular calcium-binding protein 2 (*Smoc2*). The fourth expressed moderate levels of *Col1a1* and had high expression of transcripts as Collagen XXII (*Col22a1*) and Matrilin-4 (*Matn4*). Interestingly, widely accepted tendon markers like *Scx* and Tenomodulin (*Tnmd*) that were expected to be similarly expressed in all 4 tenocyte groups, showed variable expression in each group with some markers only expressed minimally in each group^17^.

Likewise, a combined study^18^ of single-cell transcriptomics and proteomics was able to find eight distinct tendon cell sub-populations in unsupervised clustering of healthy and diseased human tendon from a variety of anatomic locations. These include endothelial cells, T cells, macrophages, and in this case, five distinct tenocyte populations. Each tenocyte group expressed different combinations of markers: (1) One group expressed microfibril-associated genes as drebrin (*DBN1)* and versican; (2) A scleraxis (*SCX+) group* co-expressing inflammatory markers; (3) A group of apolipoprotein D (*APOD+*) fibro-adipogenic progenitors; (4) Chondrogenic cells expressing genes like *COMP* and cartilage intermediate layer protein (*CILP*); (5) an integrin alpha-7 (*ITGA7+*) group of smooth muscle mesenchymal cells.

As such, single-cell ‘omics shows promise for more significant insights into the heterogenic subpopulations of tendon fibroblasts, and their roles. In addition to the benefits of an improved understanding of the biology of tendon development, these findings have the potential to contribute to the field of engineered tendon development. It is still necessary to identify if these subpopulations are present across different anatomic sites and if their distribution and expression vary within populations with different demographics. The use of new techniques such as spatial transcriptomics that combine histology and transcriptomics could improve our understanding of the nature of these differences by capturing the spatial distribution of these sub-populations.

Similarly, the use of techniques as chromatin immunoprecipitation (ChIP)-seq, methylated DNA immunoprecipitation (MeDIP)-seq, and assay for transposase-accessible chromatin (ATAC)-seq, highlight metagenomic changes that are responsible for this heterogeneity of phenotypes. These techniques can track transcription factor binding sites, histone location, accessible chromatin, DNA methylation, and other changes that allow for the elaboration of these differences. This metagenomic information could lead us to understand the role of these sub-populations and apply it to regenerative techniques.

#### Patient specific variables

**Fig. 2:**
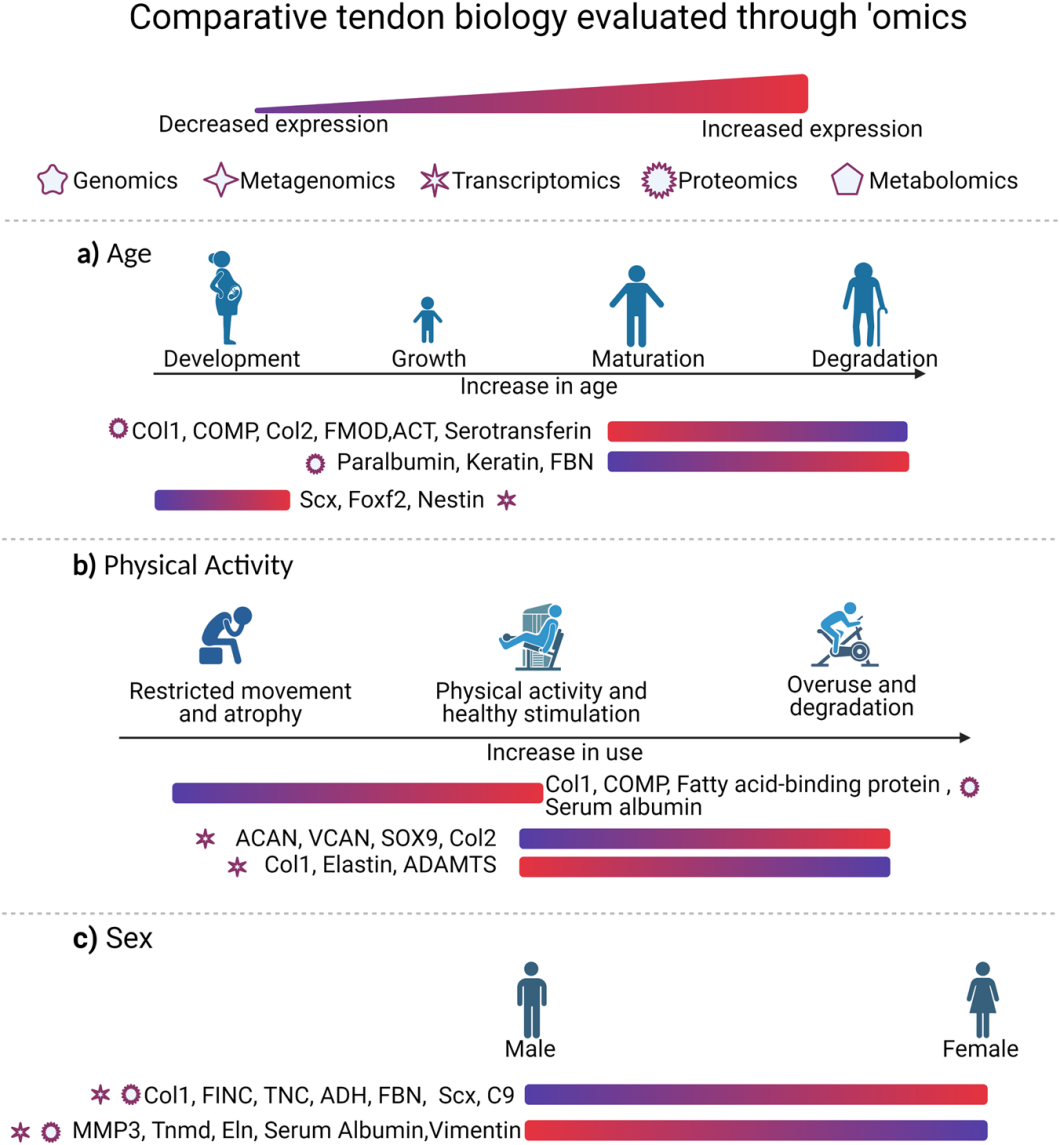
Comparative metabolic evaluation of biological differences already identified in tendon through ‘omic techniques (citations in text) according to different demographics. **a** Age, **b** physical activity and **c** sex. Collagen (COL); cartilage oligomeric matrix protein precursor (COMP); fibromodulin (FMOD); actin (ACT); fibrillin (FBN); scleraxis (Scx); forkhead box protein F2 (Foxf2); aggrecan (ACAN); versican (VCAN); transcription factor SOX-9 (SOX9); a disintegrin and metalloproteinase with thrombospondin motifs (ADAMTS) fibronectin (FINC); tenascin (TNC); alcohol dehydrogenase (ADH); complement component C9 (C9); matrix metalloproteinase 3 (MMP3); tenomodulin (Tnmd); elastin (Eln). Created in Biorender.com.

In addition to the regional structural variations present within every tendon, there are distinct demographic and other factors that influence tendon metabolisms such as sex, age, and physical activity^19^. These changes are not necessarily tied directly to tendon regeneration, but understanding these variables helps identify both the baseline, and requirements or approaches the tissue needs to take to heal correctly while preserving functionality. Figure 2 summarizes some of these important biological factors and some of the differentially expressed genes that change with them, as described below.

#### Sex

Sex is a highly relevant demographic factor for consideration in tendon biology and tendinopathy. For instance, men often have a higher incidence of Achilles tendon rupture. Recent studies have highlighted the difference in metabolic pathways such as oxidative stress, cell signaling, and immune system responses between males and females^20^. Studies^21–23^ indicate that patellar tendons in females have an abundance of alcohol dehydrogenase (*ADH1B*), complement component 9(*C9*), thrombospondin 1(*THBS1*), *TNC*, fibronectin, and clusterin, and decreased myocilin compared to males in both humans and mice. Proteins like *COL3A1* in human patellar tendon show opposite trends between transcriptomic and proteomic studies. However, these studies^21,23^ also had a limited number of biological replicates in each group (*n* = 3 male; *n* = 3 female) and may not fully represent the spectrum of variability within human populations.

Some of these apparently discordant effects of sex on tendon biology highlight the need to specifically design studies with female and male subjects as a biological variable and studies should be evaluated with this in mind. There is an additional need to understand how non-binary sex structure and medical transition influence tendon and risk of tendinopathy, to better support and treat all patient groups. These variables are frequently difficult to analyze in repositories due to the lack of patient metadata available and the inconsistency in reporting. Nonetheless, encouraging the complete reporting and annotation of multiomic data will enrich analyses and help eliminate biases in future studies.

#### Age

Proteomic studies^24–26^ in different species including humans, horses, and rats have agreed that aging (mean 24 vs. 57 years, 3 vs. 19 years, and 3 vs. 21 months respectively) results in ECM reorganization with decreases in the matricellular proteins *COL1, COMP*, *FMOD*, actin (*ACT*), and other cell adhesion proteins, and increases in cytoskeletal proteins such as keratin. These changes reduce migration and motility of cells by modifying cell–cell and cell–matrix interactions^27^. Yet, as shown in a tendon proteomic study in rats, physical activity reduces the effects of aging by increasing collagen deposition and *Comp*^26^, highlighting the importance of considering age in the context of other relevant variables.

At the other end of the aging spectrum, tendon development (E11.5 to E15.5) in mice identified *Scx* as a development marker^28,29^. These transcriptomic studies found that the transforming growth factor-beta (*TGF-β)* and mitogen-activated protein kinase *(MAPK)* pathways modulate temporal changes during development^28^ and have proposed forkhead box protein F2 (*Foxf2)* as an additional marker for mature tenocytes^29^. Similarly, Yin et al.^30^ used single-cell transcriptomics in mice to analyze tendon development (E11.5 to E14.5). Their study suggests that a subpopulation of cells in the tendon milieu during development has high *Nestin* expression, corresponding to a higher tenogenic and replication capacity than *Nestin* negative cells in which both tenogenic and cloning capacities were significantly hindered.

The integration of ‘omics in these types of studies could improve understanding of tenocyte identification, proliferation, and migration. In addition, this integration could help quantify the differences in proteins and transcripts and highlight the differences between age groups as a baseline for improved healing responses. It is relevant to note that sometimes, single ‘omics platforms may not be enough to analyze these differences. For instance, targeted epigenomics studies in this field have shown differential expression of markers. However, the influence of aging and the downstream effect the different markers have on tendons is still elusive^31^.

Overall, evaluating tendon across the lifespan from embryonic development, through post-natal maturation, and through the post-maturation aging process could allow identification of specific needs for each group with respect to healing. Similarly, tissue-engineered approaches could be modified for each group to increase regenerative responses.

#### Physical activity

Physical activity significantly affects tendon metabolism and has been studied in different tendons using different exercise protocols. A proteomic study^26^ evaluating ladder-climbing while carrying a load three times a week for 12 weeks in male rats induced differential expression in the proteome of the calcaneal tendon in 11 proteins with increases in ECM proteins including collagen 1 alpha 1 (*COL1A1*) and *COMP*. These changes were associated with decreased aging-induced downregulation of ECM proteins that cause degeneration^26^. Other differentially expressed proteins were related to cellular metabolic processes and showed that sedentary tendon specimens had a reduced capacity for fatty acid absorption, ECM remodeling, and iron transport^26^. In contrast, a downhill running (10% grade) study at 17 m/min for 1 h/day, 5 days/week for 1, 2, or 4 weeks induced clear overuse changes in the supraspinatus tendon of male rats^32^ and a transcriptomic shift to a fibrocartilage-like phenotype. Transcripts for proteins such as aggrecan (*ACAN*), versican (*VCAN*), the transcription factor *SOX-9* (*SOX9*), and collagen 2 alpha 1(*COL2A1*) were upregulated, while transcripts for proteins as *COL1A2*, elastin, A disintegrin, and metalloproteinase with thrombospondin motifs 4 (*ADAMTS-4*) were downregulated^33^. Rest reversed these overuse effects^32^.

When comparing these studies, it is not clear if the differences between pathways identified in each study are due to the type and degree of exercise, the cumulative effects of exercise history, the differential impact of exercise on the individual tendons evaluated, or due to differences in the ‘omic platform used. Since the studies were from proteomic and transcriptomic data, respectively, they represent different molecular stages, and therefore, we would not expect absolute correlation. There is likely to be a correlation between mRNA and protein content for the most highly expressed proteins, but the relationship between mRNA and protein dynamics are highly complex, and in less abundant proteins, the correlation may not exist due to greater effects of translational regulation^34^. Future studies that integrate both techniques could validate these differences and their effects and identify translational regulation as a potential therapeutic target. Regardless, these results highlight the plasticity of tendon and tendon homeostasis to mechanical stimulation.

Overall, due to the clear influence of exercise on tendon, the effects of mechanical stimuli should be considered and controlled for when possible in animal models. Similarly, obtaining information about human donor recreational or occupational physical activity in samples using human tissues could help evaluate if the changes observed are consistent with physical activity. Importantly, making this information available to other researchers through repositories is critical.

### Degeneration and healing of tendon

Understanding the difference between healthy and degenerate tendon across the disease spectrum (acute trauma, inflammation, tears, and ruptures) and throughout the healing processes is a pre-requisite for developing new therapies. Regenerative therapies must focus on improving healing outcomes and counteracting degenerative pathways. Therefore, since ‘omics techniques are able to evaluate across multiple levels of metabolism, they can identify many differences within tissues. In this regard, several ‘omics platforms have been used to evaluate healing and degenerative processes in tendons.

#### Genomic study of tendon pathology

Genomic studies relate tendinopathies to many specific polymorphisms, but SNPs identified are not consistent between tendons. For example, various SNPs in β-defensin (*DEFB1*), fibroblast growth factor 3(*FGF3*), *FGF10*, and FGF receptor *FGFR1* are significantly increased in individuals with rotator cuff disease^35,36^. In contrast, in Achilles tendon, the vascular endothelial growth factor (*VEGF*) AGG haplotype increases the risk for tendinopathy^37^. Targeted analysis of *TNC* SNPs showed a strong correlation with Achilles tendinopathy^38–40^. Regardless of the anatomic site, these genes engage in a wide array of metabolic pathways involved in remodeling, differentiation, angiogenesis, and collagen deposition.

However, these targeted studies also highlight the difficulty in finding genomic markers for a disease since the whole-exome analysis can identify more than 200,000 variants and some of these SNPs belong to genes that are not completely characterized. Therefore, the analysis of a high number of significant genetic variations is complex and may not always contribute to an improved understanding of the disease. Integration of genomic data across other ‘omic platforms could improve the differential analysis and facilitate assessing the affected metabolic pathways and the underlying SNPs responsible.

Likewise, the limited number of biological replicates within disease vs. control groups continues to be a limitation for genetic analyses. For studies of tendon, tissue availability is generally scarce from patients, and relatively few samples are available. In addition, there is often an overrepresentation of the disease’s prevalence, increasing bias in study results^38^. Small pool sizes are generally limited in diversity for ethnicity or racial background and may be disproportionately impacted by unknown confounding factors.

These challenges can sometimes produce contradicting results. Studies that evaluated previously accepted SNPs^41–45^ related to tendinopathies within a broader genomic database have been done for caspase 8 - rs1045485, a disintegrin and metalloproteinase with thrombospondin motifs 14 (*ADAMTS14)* - rs4747096, bone morphogenetic protein-4 (*BMP4*) - rs2761884, *COL5A1* - rs1134170, rs12722, rs3196378, fibrillin-rs331079, and stromelysin-1 (*MMP3*) rs591058, rs679620. When challenged against a different and larger pool of donors, there was no correlation between the previously accepted SNPs and the disease^46–48^. Still, other confounding factors, including tissue origin^48^, physical activity^36^ or ethnicity^49^, or even yet unexplored epigenetic modifications could contribute to the effects of these SNPs in specific high-risk populations. These factors could ultimately lead to over- or under-estimation of the importance of various SNPs in general populations.

#### Epigenetic study of tendon pathology

Epigenetic factors also affect pathology development. In a study of epigenetic factors in rotator cuff tear tendon biopsies at the time of surgery, matrix metalloproteinases MMP1, MMP2 MMP9, and MMP14 were negatively correlated with the regulation of microRNA (miR)-29 family members^50^. Likewise, DNA methylation regulates matrix remodeling proteins. For example, methylation differences in the *Mmp25* promoter^51^ and upstream of the *MMP11* promoter^52^ along with other genes involved in matrix remodeling, including leprecan-like Protein 2 (*Leprel2*) and forkhead box protein F1 (*Foxf1*)^51^ reiterate the importance of ECM remodeling in both tendon healing and degeneration.

Many of these studies further highlight the regulation of metabolic pathways involved in tendon biology and healing. For instance, *Foxf1*, when differentially methylated, results in upregulation of integrin β3^51^. This change controls cell senescence through activation of the TGB-β pathway^53^, resulting in tendinopathy. Yet during development, expression of *Foxf1* regulates hedgehog signaling^54^, which is critical for the development of the tendon enthesis^55^. These findings illustrate the fragile stability of metabolism and why it is necessary to not only explore a single pathway but to analyze all the possible molecular changes. In addition, since epigenetic changes have many different mechanisms (different types of non-coding RNA, histone modification, mRNA), future studies should analyze the interactions of these factors to obtain a more robust epigenetic profile of different tendon states and degrees of tendinopathy^31^.

#### Transcriptomic study of tendon pathology

Transcriptomic studies have been critical to improved understanding of tendinopathy. Supraspinatus tendon obtained during surgical repair of tears has altered ECM protein expression^56^. Perhaps unexpectedly, neurological processes are altered too. For instance, the G protein-coupled receptor protein signaling pathway is upregulated in tears, possibly regulating the immune system and inflammation response in tears^57^. Remodeling genes including several *MMPS*, lysyl oxidase (*LOX*), and *ADAM* family members are also upregulated in rotator cuff tears^58,59^.

Additional variables, including tear size are relevant for clinical outcomes. Differences in the transcriptome have been identified between small and large tears in the rotator cuff, including differential expression of collagen type -4 *(COL4), -*12*(COL12), -*14*(COL14),-* 24 *(COL24)*, and -27*(COL27)*, as well as *ACAN, TNMD, BMP5*, *TNF* receptor, and *FOXF2*^58^. Whether these differences result from underlying changes in the tendon matrix of patients that predispose to small or large tears or whether they are a result of differential loading of the tendons under these tear conditions remains unknown.

Although evaluation of gene expression is typically used on its own (either via transcriptomics, or by evaluation of expression of targeted genes) to describe a specific phenotype, transcriptomics alone may be inadequate to accurately describe of the various disease states and cannot be absolutely quantified to compare between studies. As a result, the combination of transcriptomics and proteomics could be a better approach to avoid these shortcomings.

#### Proteomic study of tendon pathology

The highly aligned and collagen-rich nature of tendon makes protein extraction and solubilization challenging. Therefore, Kharaz et al.^60^ tested the efficacy of chaotropic and detergent agents on tendons. They found that guanidine hydrochloride (GnHCl) followed by Rapigest™ extraction improved the identification of collagen species, while the use of only GnHCl improved cellular protein and proteoglycan identification. Sato et al.^61^ similarly established that treatment of tendon with GnHCl, Cyanogen bromide (CNBr), elastase, Lys-C, and trypsin allowed for complete solubilization. According to the needs of each study, different methodologies can be used, but these different methods should be considered when comparing results between studies.

Proteomic profiles indicate that immune and inflammatory response pathways are highly upregulated within tears and fibrotic tissue in tendons^56,58,59,62,63^. For example, nuclear factor-kappa B (NF-κB) has been extensively studied since it is implicated in tendon inflammation and healing. Resolution of the inflammation caused by NF-κB may be needed for tendon healing^64^. Transcriptomic studies found upregulation of NF-κB subunits RelA and p65 in fibrotic flexor tendon^65^ and these contribute to the inflammatory response^66^. In contrast however, increased activation of NF-κB through NFKB1 deletion increased collagen deposition through macrophages and myofibroblasts^67^. Similarly, interleukin 1 beta has been tied to catabolic effects on tendons and increases COMP production^68^. Proteomic profiling demonstrated that migrating macrophages heavily influence the outcome in the initial degeneration stages^69^.

Although fewer studies are available so far, the proteome of extracellular vesicles in tenocytes is enriched in collagens, TGFβ1, and cytoskeletal proteins when compared to myofibroblasts, leading the authors to conclude that these vesicles could improve collagen synthesis and ECM assembly, potentially a good resource for regenerative therapies^70^.

Combined transcriptomic and proteomic studies have highlighted other factors necessary for tendon healing. For instance, when comparing healthy and diseased tendon, single-cell transcriptomics and proteomics studies found diseased tendons had a lower proportion of *SCX*+ cells and inflammatory markers^18,71^ suggesting that resident *SCX*+ fibroblast cell populations are required for healthy tendon and restoration of these populations in diseased tendon should be further explored.

External factors, like exercise, may be similarly relevant for tendon healing as for tendon homeostasis. A study^72^ compared immobilization and early activity group in rabbits after Achilles tendon repair. Heat shock protein beta-1 (HSPB1) and annexin A2 expressed by the early activity group might contribute to healing by improving stress response.

Specific conditions are also studied through proteomics. For instance, masticatory muscle-tendon–aponeurosis hyperplasia was a poorly defined condition in which the underlying pathologies were unknown. Proteomics studies established that fibrinogen and beta-crystallin A4 were highly expressed in these hyperplastic tendons, whereas myosin light chain 4 was downregulated^73^.

#### Metabolomic study of tendon pathology

A wide variety of small metabolites are of potential interest relating to pain, mechanisms of injury, and repair in tendon. Addevico et al.^74^ showed that during recovery after surgical treatment of Achilles tendon rupture, there was a positive correlation between pyruvate concentrations 2-months after surgery and a positive general outcome after one year. Pyruvate increases the expression of antioxidants that act as an anti-fibrotic agent, minimize collagen type III, and reduce oxidative stress on collagen fibrils, therefore reducing fibrosis and inflammation.

Metabolomics study in a murine TGF-β1 Achilles tendinopathy model^75^ was able to identify relevant species for tendon regeneration: creatinine, lactic acid, pyroglutamic acid, and D-chiro-inositol. The study concluded that creatine supplementation for tendon is a viable tool for healing, and that the myo-inositol pathway alteration may be responsible for chondroid deposition in tendinopathy. In addition, both injured and healthy samples were lipid rich and phosphatidylcholines and lysophospholipids were differentially altered with injury. These results suggested that these lipid species may have a role in lubrication and should be studied further.

Similarly, Flück et al.^76^ used a combined transcriptomic and lipidomic approach to improve understanding of fatty infiltration of infraspinatus muscle following infraspinatus tendon detachment and repair, and the mechanism of the beneficial response to the anabolic steroid, nandrolone in preventing muscle to fat transformation. A follow-up study^77^, evaluated early metabolic processes that cause this fatty atrophy by studying mitochondrial dysfunction and its treatment with L-carnitine. Tendon detachment led to downregulation of mitochondrial transcripts, decreasing oxidative phosphorylation, and promoting lipid accumulation. L-carnitine, used in other animal models to activate mitochondrial metabolism was not able to prevent these changes. Although not directly related to tendon healing, these studies improve the understanding of relevant factors associated with tendon healing that could impact the mechanical and local adipokine environment following repair.

In summary, there are still many gaps that need to be addressed concerning tendon healing that the use of ‘omics can address. It is essential to identify if the changes in diseased tissues result from the pathology or reflect a predisposition for the process that leads to clinical tendinopathy. Furthermore, it is important to expand current studies of single-cell characterization to identify if the subpopulations of tendon fibroblasts identified to date are also present in diseased and healing tissues and to understand how their distribution changes through tendon remodeling.

### Tendon phenotype in vitro

Cell seeding into constructs in vitro is a standard procedure for developing and testing tissue engineering approaches, but a number of variables can profoundly impact outcomes. Work with stem and progenitor cells and tendon fibroblasts can promote the differentiation into different lineages or induce dedifferentiation, respectively^78,79^. As such, aberrant phenotypes may be produced and may ultimately cause undesired side effects or impaired function^80,81^. Therefore, adequate characterization of tendon phenotypes in vitro is an essential step in the bench-top evaluation of tissue-engineered approaches.

#### Tendon phenotype evaluation and definition

Multiple markers are widely used to define tendon phenotypes. These markers were identified with diverse techniques, and their use is supported by transcriptomic profiling: *TNMD*, *TNC*, *SXC*, decorin (*DCN)*, biglycan (*BGN)*, *ACAN*, *COMP*, homeobox protein Mohawk (*MKX)*, *COL1A1*, *COL3A1*, *COL1A6*, early growth response protein *(EGR1*), *FMOD*, *MMP3*, *MMP1*, and *ELN*^28,82–89^. However, despite these findings, each of these markers are also common in other connective tissues and is not unique marker for tendon^90^. Typically, transcription levels of a few of these markers are used to evaluate tendon phenotype. Still, tissue-engineered approaches have shown that when comparing tendon constructs to other connective tissue constructs, the transcriptomic differences between native tissues are no longer present even when there is the presence of the tendon markers^90^. These results indicate that the tendon phenotype cannot be defined only by the presence or upregulation of tendon markers.

The contrast between tendon and other related connective tissues could be used to define better markers for tendon phenotype^90^. For instance, the anterior cruciate ligament is enriched in cartilage-like proteins like *COL2A1*, aggrecan, and chondroadherin^91^ compared to the patellar tendon. The patellar tendon instead is enriched in *ELN, TNC, COMP, COL3A3*, and *COL12*^23,91^. Similarly, when analyzing the transcriptome, the enthesis shows enrichment of biological processes like proteoglycan biosynthesis, chondrocyte differentiation, collagen organization, ossification, and angiogenesis in comparison to tendon, and has higher levels of *COL9*, *COL2*, and *MMP13*^92^ transcripts. In this regard, characterization of tendons in all stages and conditions through multiomics could identify if there is a need to quantify a ratio between these markers, if absolute quantification methods are more appropriate, and even if these markers are adequate, or if there are other relevant markers that have been overlooked so far.

#### Stem cells derived tenocyte differentiation

Characterization of differentiation processes is critical to define how close the phenotypes obtained in vitro are to native tissue. No genomic studies have examined differentiation, presumably because the genome is not expected to change with differentiation. Of potential relevance to translation however, there are no studies to examine how the genome or variants influence tenogenic differentiation.

Stem cell source is relevant for in vitro studies. Epigenetic factors such as DNA methylation and miRNA, along with protein translation, are differentially regulated in older populations^93,94^, and as a result, cells could become more, or less responsive to environmental cues. Consequently, the age of stem cell donors is a critical factor for regeneration.

Yet age is not the only relevant factor for the regulation of plasticity, although many of these other factors remain to be thoroughly evaluated. Based on work by Harvey et al.^95^, that showed a key role for regulation of glycolytic pathways in epigenetic regulation of embryonic stem cell pluripotency via histone acetylation, Oestreich et al.^96^ speculated this as a potential mechanism by which maternal obesity could alter metabolic plasticity for future generations, and impair stem cell function.

Transcriptomic studies have explored tendon differentiation more frequently than epigenetic studies to date. As mentioned before, tendon development studies in *Scx-GFP* transgenic mice have identified *Foxf2* as a tendon marker and the *TGF-β* and *MAPK* pathways as the most modified during development^28,29^. There are also differences between cells programmed to a tendon lineage and those already present in native tissue. In this regard, cells differentiated towards a tendon lineage usually lack the muscle-like characteristics of native tendon^79,97^. Engineered tendon constructs fabricated from a non-fiber based fibrin gel system have greater cellularity than native tissues and lower collagen and sGAG content^91^, perhaps representing a more immature phenotype. However, substantial knowledge gaps remain in understanding how stem cells differentiate towards tendon cell lineages in response to biomaterials.

Integration of transcriptomic and proteomic profiling identified that different stem cell populations distinctly favor different biological processes. For example, the cell cycle is more dominant in embryonic stem cells, whereas extracellular remodeling is more dominant in bone marrow-derived mesenchymal stem cells^98^. As a result, stem cell origin influences the proteome^90,97,99–101^, demonstrating that cell differentiation across different cell sources should be fully characterized during the development of regenerative medicine approaches. It is essential to evaluate through ‘omics the differences between tendon-derived cells, tenocytes obtained from different stem cells, and any other source cell against the native tendon to understand the differences between them and the derived metabolic changes that may affect regeneration or functionality.

#### Quantification methods for studies

Beyond basic ‘omic profiling, there is still no consensus as to the most appropriate way to evaluate tendon phenotypes, although as always, this likely depends on the research question. For example, in some cases, comparison of the proteome between experimental groups may be adequate, yet, absolute quantification methods may be preferred in some cases. However, wherever possible, absolute quantification facilitates comparison between studies and research groups. Similarly, the integration of different ‘omics techniques as genomics and transcriptomics with quantitative proteomics and metabolomics can be used as an unbiased standard to compare samples and studies. In addition, consistent use of healthy native tissue or harvested tenocytes as a positive control are highly encouraged in evaluation all tissue-engineered approaches.

### Regenerative and therapeutic approaches in vitro and in vivo

Existing knowledge about tendon metabolism and healing can be used to improve tissue-engineered approaches but deep-profiling of engineered approaches provides the opportunity to (1) improve understanding of the diverse variables in tendon biology; (2) follow the regeneration process over time; and (3) identify issues that need to be addressed to improve the outcome. For tendon, the evaluation of these approaches through ‘omic techniques has been restricted to proteomics to date, and has evaluated the efficacy of materials for constructs and their overall performance, as well as external variables affecting the healing process.

#### Tissue-engineered tendon construct evaluation

Kharaz et al.^91^ were the first to identify the differences between engineered tendon and ligament constructs and native tissues. This study showed that the nuances that distinguish these two tissues in vivo were lost in the engineered approach^91^. Clustering of native tendon, ligament, and tendon and ligament constructs showed three different groups: one for native tendon, one for ligament, and one for tendon and ligament constructs. These differences are explained by increased ECM remodeling in the constructs. Pathways involved in proteolytic activity, biodegradation metabolism, and signaling proteins were enriched, while ECM protein content was decreased compared to native tissue on day 14^91^. These results highlight the need to define better when engineered tendon can be considered tendon tissue^102^ and could be far longer than the current time points evaluated in vitro, particularly in light of the long post-natal tendon maturation period. Regardless, this knowledge is relevant to determine the best time for implantation after pre-culture in a clinical setting, or alternatively suggest the time needed for cell infiltration, differentiation, and maturation if biomaterials are implanted and seeded with cells at the time of surgery. Answering the ‘When is good enough?’ question will also inform post-operative rehabilitation protocols.

#### Biomaterial evaluation

‘Omics studies have improved understanding of the immune response to biomaterials. For instance, evaluation of autologous stem cells and *BMP12* seeded on a nanofiber/fibrin-based PLGA scaffold insertion in vivo showed the scaffolds increased proteins related to inflammatory and immune response, stress response, and decreased ECM proteins^103^. These results were unexpected based on previous studies from the same group, which showed that fibrin and *BMP12* did not exacerbate the immune response.

Other studies^104^ have used proteomics to look for inflammatory pathway upregulation as a means of identifying potential safety problems with engineered approaches that could impair in vivo repair. Delivery of connective tissue growth factor (*CTGF)* through sutures in a canine flexor tendon injury model found no difference in inflammatory protein levels compared to sutures without *CTGF*^104^, suggesting that *CTGF* is not pro-inflammatory or detrimental when incorporated into engineered strategies in vivo.

Tendon development in constructs made of collagen and fibrin, when compared to embryonic tendons of diverse ages, showed that fibrin and collagen constructs did not induce the majority of upregulated genes in embryonic tendons^105^, with the conclusion that the fibroblasts on collagen samples may undergo senescence. Fibrin constructs showed more resemblance to embryonic tendons indicating that healing may improve when cells encounter fibrin-based scaffolds, and later produce collagen.

These studies illustrate how ‘omics improve understanding of the impact of relatively simple changes to scaffolds. They also highlight the need to standardize characterization, so it is possible to follow changes and find differences in pathways resulting in aberrant outcomes.

#### External variables effects on tissue-engineering evaluation

Mechanical cues are critical to tendon remodeling and differentiation. As such, bioreactors are frequently used in vitro to mimic the same remodeling processes that occur in vivo and to understand the mechanisms of tendon response to cyclical loading. In this regard, differentially expressed proteins correlate with pathways of mechanotransduction and ECM remodeling and deposition^106–109^. Further, proteomic studies have highlighted the role of mechano-activated channels in tendon, including identifying the cystic fibrosis transmembrane conductance regulator (*CFTR*) as critical in tendon regeneration since dysfunctional *CFTR* mice exhibited weakened tendons with less matrix formation and uneven cell arrangement and distribution^110^. Overall, these previous studies demonstrate that ‘omics platforms improve characterization of tendon regeneration and allow for more thorough and informed conclusions about tendon biology, novel targets, therapies, biological processes, and ongoing challenges for the field to work on.

### Bioinformatics for ‘omics integration

Combining’omics techniques is crucial to understanding which pathways differ between experimental groups. The integration of multiple techniques can enhance the multiplicity of the data, and as a result, identify relevant pathways that would otherwise be overlooked. Yet, tendon studies with multiple ‘omic techniques are scarce. Nevertheless, other fields have clearly demonstrated the value of data integration across ‘omics platforms.

Cancer research is the field where multi-omics integration has been most employed. For instance, a study by Zhang et al.^111^ on ovarian cancer samples integrated transcriptomics, proteomics, and epigenomics (DNA methylation and miRNA sequencing), discovered 9 different subtypes of cancer. Each type was associated with activation or suppression of immunoactivity, hormone processes, mesenchymal development, or the MAPK signaling pathway, suggesting possible mechanisms for ovarian cancer. Like these studies, others have also used the integration of different ‘omics to perform clustering and illuminate different pathways related to disease^112–117^.

In contrast, other studies, instead of analyzing the pathways, use multi-omics to predict prognosis. For instance, Chaudhary et al.^118^ integrated transcriptomics and epigenomics (DNA methylation and miRNA sequencing), along with clinical data to identify survival in liver cancer. Similarly, a study^119^ integrated mRNA, miRNA, proteomics (profiled and targeted), and metabolomics (profiled and targeted), through similarity network fusion. This study was able to identify chronic obstructive pulmonary disease in small pools with 95% accuracy, even with confounding factors as smokers and non-smokers in the groups.

In order to reach this insight, all the data must be associated with a function. Standardized ‘vocabularies’ allow for annotation of the genes and gene products according to their associated biological processes, cellular components, and molecular functions via extensive databases. These databases allow for understanding families, interactions, modifications, enzymatic relationships, biomolecular interactions and pathways, disease associations, and much more.

Yet, there are numerous challenges in bioinformatics and integration across ‘omic platforms. For instance, data analysis is complicated because of the different quantification and normalization techniques. In addition, due to different protocols, concentrations of input biological material and technique detection limits may vary. As a result, each dataset has to go through quality control (outlier detection, missing data imputation, normalization) separately, before integration.

**Fig. 3:**
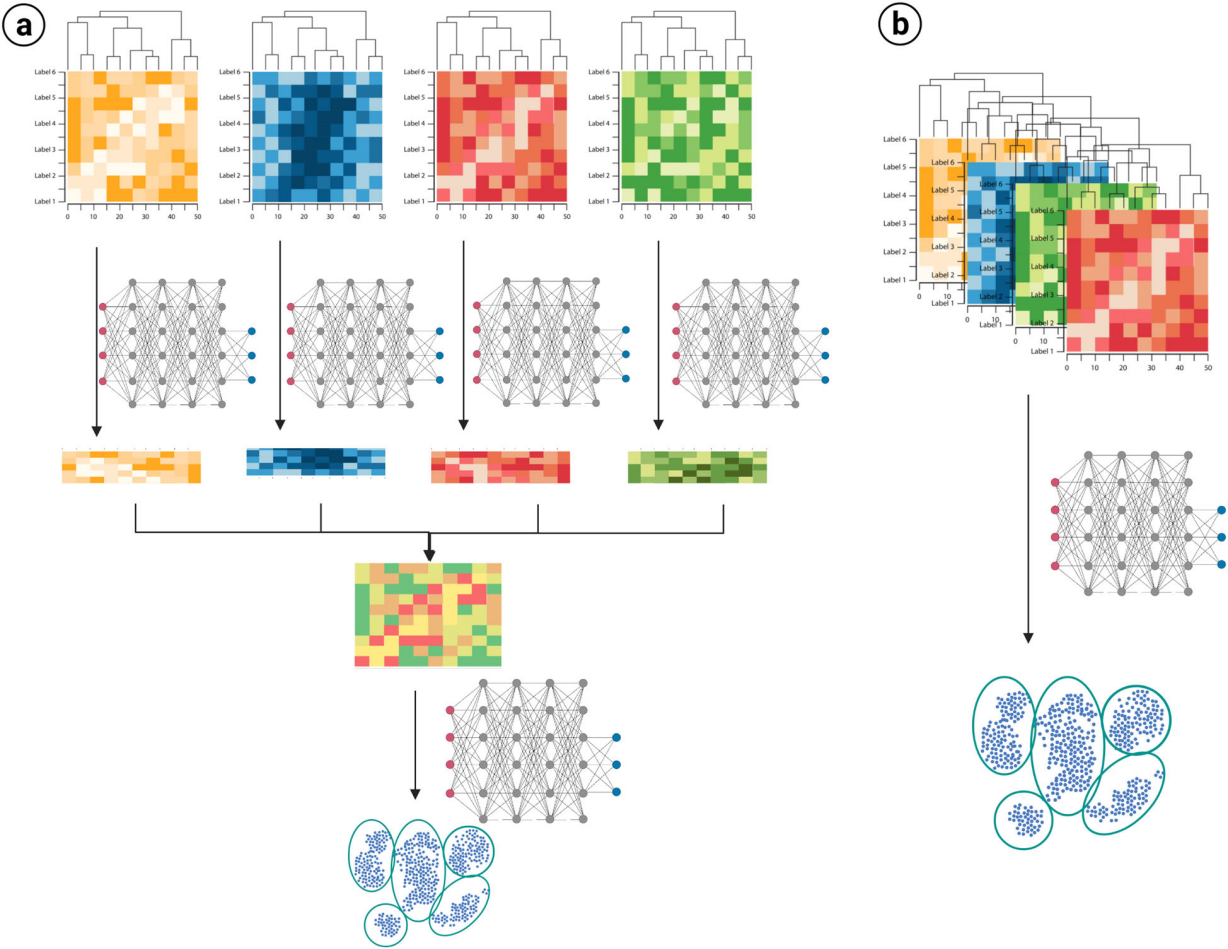
Algorithmic approaches to multiomic integration. **a** Late integration applies transformation or modeling for each of the different ‘omic datasets, and these are later integrated to construct a model. **b** Early integration concatenates all the different datasets and constructs a single model based on all the data. Clustering models and transformations (presented only as deep networks) can vary according to the approach and algorithm selected. Created in Biorender.com.

For integration, multiple different methods are available. Initially, the integration process can be classified using algorithmic approaches. As shown in Fig. 3, there is early and late integration. In early integration the ‘omics datasets are appended into a single one and then clustering algorithms applied to that information. Alternatively, in late integration each ‘omic dataset is clustered or transformed and then this model is integrated to build a new, final model.

Multiple algorithms can be used to generate these models. Simple machine learning models, as Expectation Maximization, K-clustering are often used for integration. In addition, similarity-based methods as spectral clustering, similarity network fusion, multiple kernel learning have been used in the past as well. Tensor construction, and other matrix algebra are used to transform the data and decrease the dimensions of the data. If biological knowledge can be included in the model, statistical methods are used. Finally, deep learning algorithms are often used since it includes unsupervised feature learning and then clustering.

The selection of the process and algorithm to integrate and model the data are case dependent. All the various algorithms can be considered according to the types of ‘omics data to integrate, the number of features, the information of the system, the volume of the data, the computational resources, and the available software. Therefore, consideration of different models and even comparative evaluation should be done, since the most robust output would be expected to withstand more than a single model or comparison.

## Conclusion and future directions

**Fig. 4:**
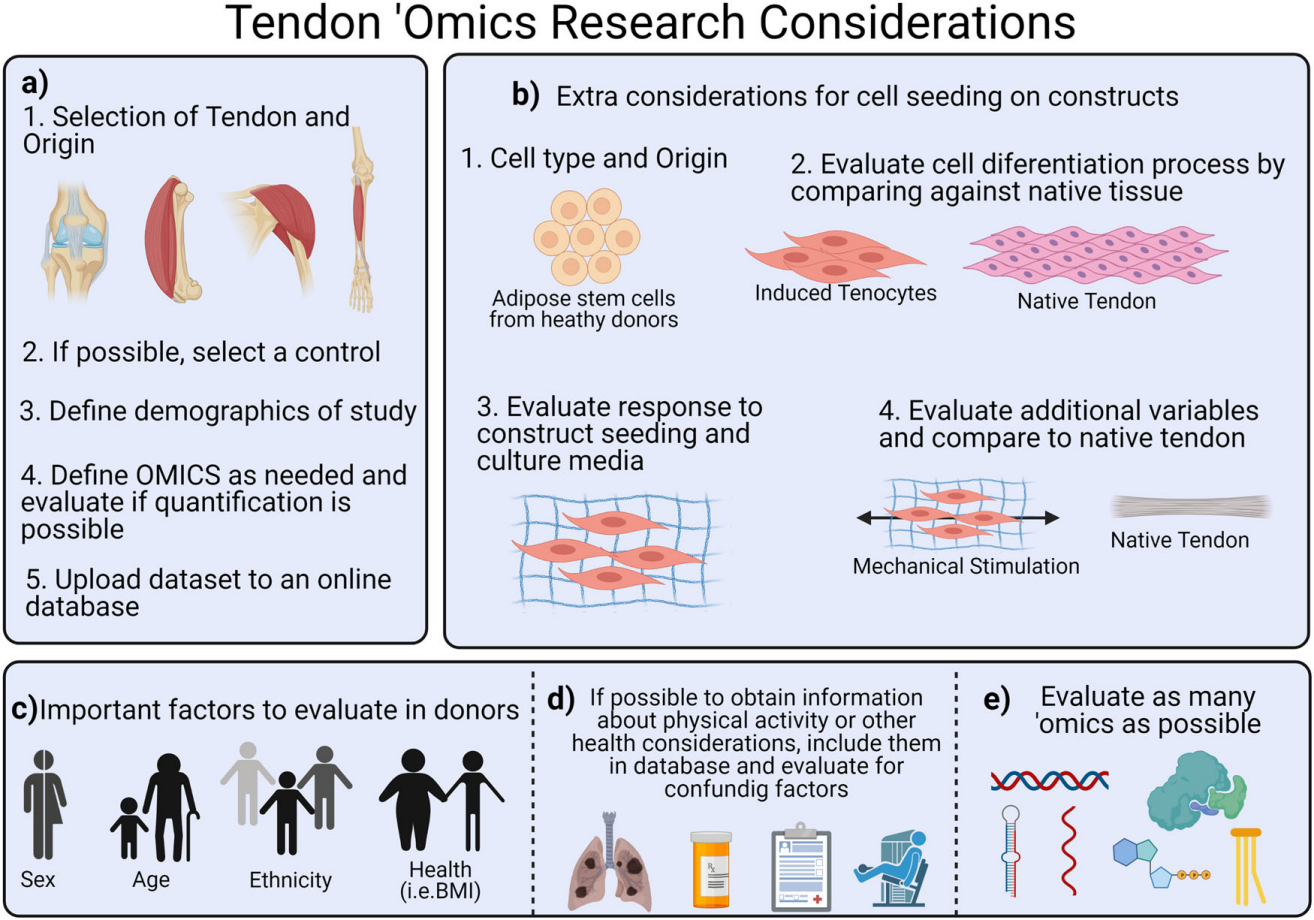
Considerations for tendon ‘omics research. **a** For all studies involving tendon tissue, it is important to define a single type of tendon and origin, and correctly define a control. **b** In case of cell seeding for tissue engineering, the cell type and origin of the cells must be evaluated carefully considering various extrinsic and intrinsic factors. If tenocytes are induced from stem cells, the phenotype should be evaluated by comparing it to the native tendon through ‘omics. In addition, if the objective of the study is to evaluate a construct, the effect of the construct should be studied initially, and then, additional variables of interest can be evaluated and compared to native tendon as a control. **c** For all studies, it is important to define the demographic factors to collect samples (sex, age, ethnicity, and health) according to the needs of the study and availability. **d** When possible, all medical information that may be provided should be updated to an online database**. e** The goal of the study defines the ‘omic techniques to use, yet we recommend using as many techniques as feasible. Created in Biorender.com.

This review of the literature on the ‘omics field regarding tendon tissue engineering and regeneration sheds light on how much the various ‘omics platforms have contributed to understanding the processes driving tendon cell behavior. However, as always, the field continues to expand. Therefore, we propose multiple considerations for future studies (Fig. 4). It is relevant that future research considers specific tendons, the use of positive and where relevant, negative controls, and the evaluation of relevant processes and factors before the experiment is completed. In addition, biological factors of relevance should be taken into account, and the metadata and ‘omics datasets should be shared on public databases.

There are some important gaps in basic science knowledge of tendon biology, pathology, healing, and regeneration when filled will improve tissue engineering, orthobiologic, and other therapeutic approaches, approaches which themselves could be enhanced through evaluation with multiomic platforms. For instance, the complexity of tendon hierarchical structure, and the broad spectrum of cells involved in tendon degeneration call for a better understanding of tendon pathologies across the spectrum in various anatomic sites.

Similarly, there is a need to better define tendons, understand the requirements for tendon differentiation and regeneration, and the basic conditions to standardize the evaluation of therapies (e.g., markers, concentration profiles, cellularization, matrix to cell ratio). Finally, the progression of metabolic and molecular changes should also be studied through the progression of tendinopathy to establish which of these changes reflect a predisposition to disease and which are the effects of the disease itself.

**Fig. 5:**
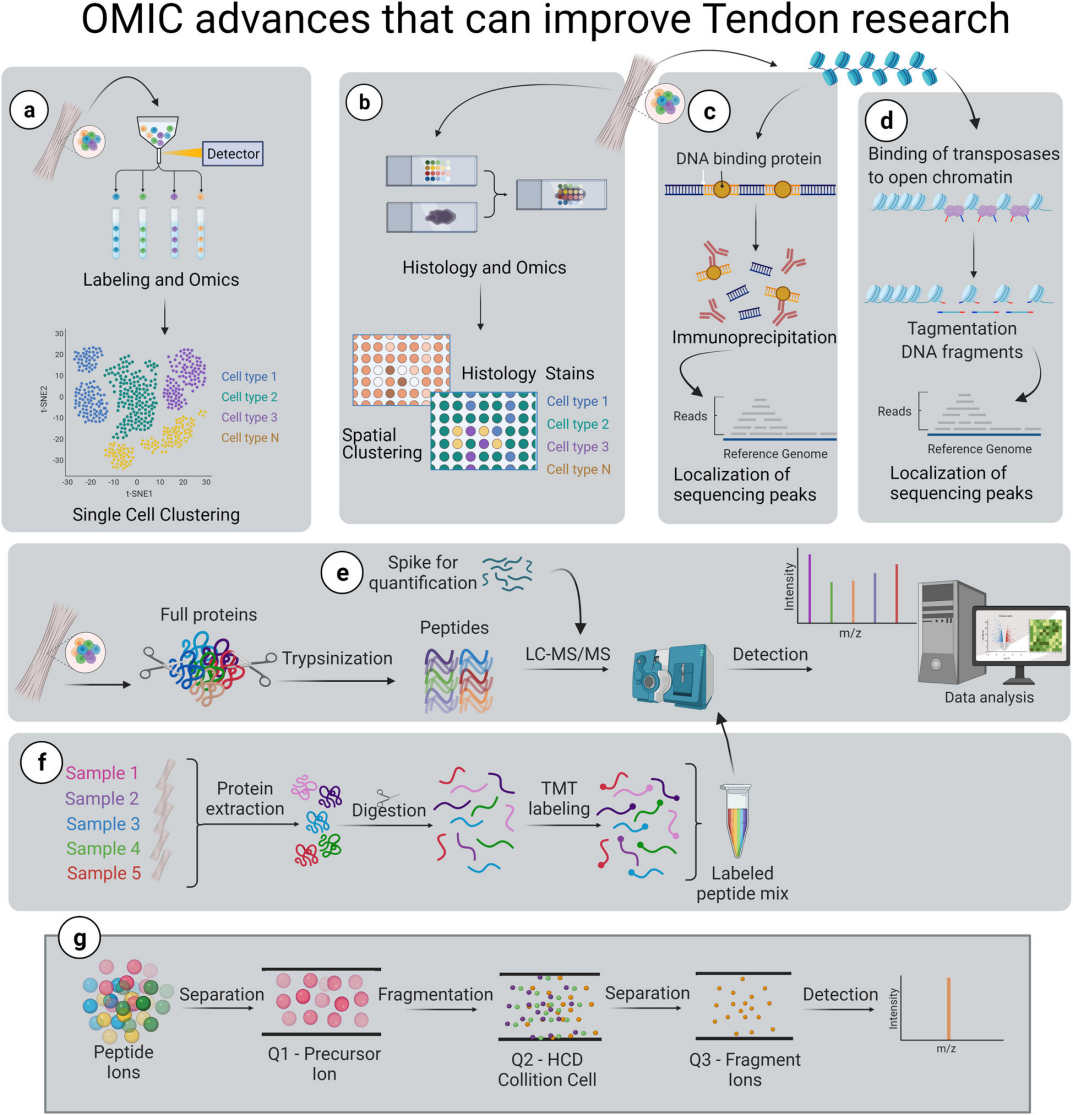
‘Omic advances that can improve capabilities of tendon research. **a** Single-cell ‘omics allows for evaluation of differential expression of genes by cell, or sub-cellular component, and then identify clusters. **b** Spatial clustering ‘omics allows for the evaluation of samples in slides that allow for histology staining and identification of genetic material by location, in order to obtain an image of differential gene expression. **c** Immunoprecipitation sequencing allows the identification of regions through the chromatin that currently attach any protein (i.e., histones, transcription factors) and permits the understanding of transcriptomic changes through metagenomics. **d** Assay for transposase-accessible chromatin sequencing allows to identify the accessible areas of the DNA that allow different phenotype differentiation. **e** Proteomics allows for absolute quantification by spiking samples with known or labeled peptides. **f** Similarly, Tandem Mass Tag labeling allows for the evaluation of multiple samples simultaneously. **g** The proteomic analysis allows for a selection of peptides and fragments that can be quantified in order to define a specific phenotype. Created in Biorender.com.

These gaps in knowledge and the complexity of tendon biology increase the difficulty of producing fully functional approaches for tendon healing. Therefore, we recommend the use of ‘omic techniques that are able to fill these gaps efficiently (Fig. 5). Overall, the tendon field has yet to widely adopt these technological advances. Nevertheless, the information that could be gained by making use of multiple ‘omic platforms is invaluable. We would be able to better define different populations in heterogenous tissues, identify transcriptomic changes and their origins, and quantify and evaluate multiple samples simultaneously as well as generate reliable systems and high throughput screening panels to improve phenotype evaluation.

These advances in technology and current work using ‘omics platforms provide insight regarding the substantial promise of this field to contribute substantially to fill these gaps towards the design, implementation, and evaluation of tissue-engineered therapies for tendon repair.
